# Prehospital Ultrasound to Identify Endotracheal Tube Dislodgment in an Infant: A Case Report

**DOI:** 10.5811/cpcem.63870

**Published:** 2026-09-20

**Authors:** Nicholas A. Wright, Ryan Lucas, Peyton Bailey

**Affiliations:** * Edward Via College of Osteopathic Medicine, Blacksburg, Virginia; † Christiansburg Emergency Medical Services, Christiansburg, Virginia

**Keywords:** point-of-care ultrasound, airway management, out-of-hospital cardiac arrest, emergency medical services, pediatric resuscitation

## Abstract

**Introduction:**

Point-of-care ultrasound (POCUS) is well established as a useful adjunct for airway management in both adult and pediatric patients, including confirmation of successful endotracheal intubation. While suprasternal ultrasound of the trachea is the most described method for confirming endotracheal tube (ETT) placement, a focused lung exam can be used to rapidly identify appropriate ventilation in otherwise apneic patients.

**Case Report:**

This case involves a one-month-old male infant who experienced an out-of-hospital cardiac arrest secondary to suspected sudden infant death syndrome. During resuscitation, an accidental extubation occurred after an attempt at securing the ETT with a commercial pediatric tube holder. Loss of end-tidal carbon dioxide (ETCO_2_) waveform was recognized immediately; however, emergency medical services clinicians questioned the device’s accuracy due to earlier failures of the ETCO_2_ monitor and the ETT remaining secured at an expected depth. A rapid POCUS lung examination demonstrated absence of bilateral lung sliding. The patient was immediately extubated, revealing a kinked ETT in the oropharynx with the distal tip positioned above the vocal cords. A supraglottic airway device (SGA) was placed with a successful return of ETCO_2_ waveform, followed shortly by successful reintubation because of difficulty ventilating through the SGA.

**Conclusion:**

Lung ultrasound is a rapid and effective alternative for identifying ETT dislodgment when conventional verification methods are unreliable or unavailable. As ultrasound becomes increasingly available in prehospital and resource-limited settings, it may be effectively used by nonphysician clinicians to confirm airway placement and detect airway complications.

## INTRODUCTION

Point-of-care ultrasound (POCUS) is an increasingly used adjunct for airway management in both adult and pediatric patients. Established applications include confirmation of successful endotracheal intubation, detection of esophageal intubation, and assessment of adequate lung ventilation.^1^,^2^ Although suprasternal ultrasound of the trachea is the most described sonographic method for confirming endotracheal tube (ETT) placement, lung ultrasound can also be used to provide rapid confirmation of effective ventilation through visualization of bilateral lung sliding in an otherwise apneic patient.^3^,^4^

Prehospital access to ultrasound continues to expand, offering a valuable method for identifying airway complications, particularly in resource-limited environments when standard confirmation techniques fail. We report a case of accidental ETT dislodgement during the out-of-hospital cardiac arrest of an infant in which lung POCUS enabled rapid diagnosis when conventional confirmation methods were unreliable.

## CASE REPORT

In July 2025, emergency medical services (EMS) responded to an emergency call for a “newborn not breathing.” On arrival, a law enforcement officer was performing cardiopulmonary resuscitation on a 1-month-old male infant who was pulseless and apneic. The arrest was presumed to be secondary to sudden infant death syndrome (SIDS), because a parent was co-sleeping with the patient and awoke to find him unresponsive. It was unknown how long the patient had been in arrest; however, no immediate contraindications to resuscitation were identified.

Resuscitation was initiated in accordance with the EMS agency’s standards of care and American Heart Association guidelines. Compressions and ventilations were performed at a 3:1 ratio until an appropriately sized supraglottic airway device (SGA) was placed. Following intraosseous (IO) access epinephrine (0.01 mg/kg) was administered at appropriate intervals. At some point during the resuscitation, it was noted that the initial IO had infiltrated, and a second IO was successfully placed in the left distal femur.

Following SGA placement, it was noted that there was poor compliance with the device and copious amounts of frothy sputum were accumulating in the device and the bag-valve-mask (BVM). Despite aggressive suctioning, compliance did not improve. The decision was made to perform ET intubation with a 3.0-mm uncuffed ETT. Intubation was successful, confirmed by continuous waveform end-tidal carbon dioxide (ETCO_2_) consistent with BVM ventilations, absence of epigastric sounds, and presence of bilateral lung sounds. Shortly thereafter, secretions occluded the ETCO_2_ sampling line, causing intermittent loss of waveform. The device was exchanged for a new one, which corrected the problem.

During securement of the ETT with a commercial pediatric tube holder, the ETT was inadvertently withdrawn from the oropharynx. The clinician readvanced the ETT, suspecting that the tube had not been fully withdrawn from the trachea, but a sudden loss of ETCO_2_ occurred, raising concern for ETT dislodgment. Some clinicians attributed waveform loss to recurrent ETCO_2_ device occlusion, and secretions were once again visualized in the sample tubing. Lung sounds were auscultated and interpreted as “diminished” by the clinician.

Given the uncertainty of conventional confirmation methods and immediate availability of an ultrasound device, a rapid bilateral anterior thoracic examination was performed during the next rhythm check. The ultrasound examination along the midclavicular lines demonstrated an absence of bilateral lung sliding (Video).

The patient was immediately extubated, and direct visualization of the oropharynx revealed a kinked ETT with the distal tip positioned above the vocal cords, most likely caused by blind readvancement of the ETT. The SGA was immediately reinserted. Ventilation through the SGA once again demonstrated poor compliance and inconsistent ETCO_2_ waveforms, prompting repeat intubation. A second clinician successfully performed ETI using a 3.5 mm uncuffed ETT. Waveform ETCO_2_ returned and remained consistent with BVM ventilations. Chest compressions were not halted for either intubation attempt.

*CPC-EM Capsule*

What do we already know about this clinical entity?

*Point-of-care ultrasound (POCUS) is an established adjunct for confirming endotracheal tube (ETT) placement, though infant and prehospital data remain limited.*

What makes this presentation of disease reportable?

*This case highlights the use of prehospital ultrasound to rapidly diagnose accidental ETT dislodgement in a one-month-old infant in cardiac arrest.*

What is the major learning point?

*Lung ultrasound can be used by nonphysician clinicians to rapidly detect tube dislodgment when conventional confirmation methods are unreliable.*

How might this improve emergency medicine practice?

*Prehospital POCUS may improve recognition of ETT dislodgment when conventional confirmation methods are unreliable.*

After 40 minutes of advanced on-scene resuscitation without return of spontaneous circulation, the closest emergency department (ED) was contacted regarding patient disposition. The attending physician directed the continued administration of epinephrine and transport of the patient. The patient was moved to the ambulance and transported without complication. Once at the receiving facility, the ED staff continued the resuscitation for a brief period prior to terminating efforts.

The medical examiner determined the cause of death to be SIDS and identified a tracheal injury assumed to have been caused by the mother compressing the patient’s neck while co-sleeping. This may have contributed to the observed ventilation difficulty while the SGA was in place.

## DISCUSSION

Rapid confirmation of correct ETT placement is critical whenever ETI is performed but particularly in infants where any amount of hypoxia drastically worsens outcomes.^5^ Continuous waveform ETCO_2_ monitoring is considered the gold standard of confirming tracheal intubation and for detecting misplacement; however, unreliable readings may occur due to device failure or occluded sample tubing. It has also been established that other methods of ETI confirmation, such as auscultation of lung sounds and visualization of condensation in the ETT, are less effective and not sufficiently reliable to confirm correct ETT placement.^6^,^7^

In prehospital and austere environments; limited resources, volatile scenes, and frequent staffing shortages pose challenges to clinicians in ensuring continuous confirmation of ETT placement. Facilitating patient movement or performing tasks with a solo clinician that are traditionally performed with several people in the in-hospital environment (such as ETT securement) poses a serious risk for ETT dislodgement. When combined with the limitations of continuous monitoring in the prehospital environment—such as lung auscultation or colorimetric end-tidal capnometry, unrecognized ETT dislodgment is a known risk. Even though the clinicians had access to the waveform ETCO_2_ capnography, it still proved to be ineffective because of suspected equipment failure. These challenges are amplified in pediatric ETI due to the high-acuity, low-frequency nature of the procedure.

In recent years, decrease in cost and widespread access to educational materials has resulted in a substantial increase in POCUS implementation throughout EMS systems in the United States. As previously discussed, ultrasound has been established as a reasonable and accepted method to verify ETT placement in both adult and pediatric patients. While most of the studies in this area focus on direct visualization of the ETT within the trachea for confirmation, some have also described using thoracic ultrasound to visualize pleural sliding as evidence of effective ventilation.^8^ One study found that “use of the ultrasound-guided lung sliding sign was more accurate than auscultation” for ETT positioning.^9^

With minimal training, non-physician prehospital clinicians can accurately determine the presence or absence of lung-sliding using thoracic ultrasound.^10^,^11^ Thoracic ultrasound is a simple, cost-effective, and non-invasive procedure that, in the setting of emergent pediatric intubation and equipment failure, may be utilized as an adjunct assessment technique to confirm sustained tracheal placement of an ETT. Although well described in hospital settings, reports of prehospital clinicians using thoracic ultrasound in infants to verify ETT position are lacking. This case illustrates how lung POCUS can rapidly identify ETT dislodgement when traditional methods fail or the environment is not conducive to traditional confirmation methods.

## CONCLUSION

This case outlines three important points: 1) Thoracic-lung ultrasound is a rapid and effective alternative for identifying ETT dislodgment in pediatric patients when conventional verification methods are unreliable or unavailable; 2) in resource-limited or high-acuity environments, ultrasound may improve airway safety; and 3) as ultrasound becomes increasingly available in prehospital and austere settings, it may be effectively used by nonphysician clinicians to confirm airway placement and detect airway complications. Additionally, while suprasternal tracheal sonography has been established as an effective method for confirming endotracheal tube intubation, many clinicians already perform thoracic ultrasound in routine practice, and these same methods can be readily applied to airway management.

## Supplementary Information

Video. Bilateral anterior thoracic lung exam performed in the sagittal plane along the midclavicular lines. The initial window is of the patient’s left thorax, followed by the right. Lung sliding is absent bilaterally (arrows).
